# Understanding Individual Barriers to HIV Testing Among Undergraduate University Students: Results From a Cross-Sectional Study in Italy

**DOI:** 10.3389/fmed.2022.882125

**Published:** 2022-04-19

**Authors:** Francesca Licata, Silvia Angelillo, Carmelo Giuseppe Angelo Nobile, Gianfranco Di Gennaro, Aida Bianco

**Affiliations:** ^1^Department of Health Sciences, School of Medicine, University of Catanzaro “Magna Græcia”, Catanzaro, Italy; ^2^Department of Pharmacy, Health and Nutritional Sciences, University of Calabria, Cosenza, Italy

**Keywords:** HIV testing, sexual behaviors, high-risk behaviors, university student, Italy

## Abstract

**Background:**

In Europe during 2019, just over half of those with HIV were diagnosed at a late stage of infection. Even though HIV testing is crucial for all strategies related to care, prevention and treatment of HIV/AIDS, we hypothesized that it is less practiced among university students, and, therefore, this study aimed to assess the uptake and factors associated with HIV testing in southern part of Italy.

**Methods:**

A cross-sectional study was conducted from 1st to 31st July 2020 among undergraduate university students aged 18–29 years. Data were collected through an anonymous online questionnaire and included questions on socio-demographic and sexual history characteristics, knowledge and attitudes toward HIV infection, sexual and testing behaviors, and sources of information about HIV.

**Findings:**

Among 1007 students, 41.5 and 54.7% knew that in Italy the test for early detection of HIV infection has not to be prescribed by a physician and that it is provided to anyone free of charge, respectively. Only 16.2% of the eligible students reported having ever tested for HIV and a very similar proportion (17.8%) was displayed among those who reported risky sexual behaviors. The multiple logistic regression analysis results indicated that the strongest predictor of HIV testing was attending medical or life sciences majors.

**Interpretation:**

The uptake of HIV testing was low among Italian university students. Effective strategies to increase HIV testing and diagnoses have to aim at overarching individual barriers, such as lack of knowledge about information around the test itself.

**Funding:**

This research did not receive any specific grant from funding agencies in the public, commercial, or not-for-profit sectors.

## Introduction

The number of newly reported HIV diagnoses and the estimated number of new HIV infections in the World Health Organization (WHO) European Region show that more people have become infected with HIV over the last decade than have been diagnosed, indicating that the number of people living with undiagnosed HIV is increasing in this Region (1). In 2020, just over half (51%) of those with HIV in the countries of European Union/European Economic Area were diagnosed at a late stage of infection (2), when the immune system has already started to fail. HIV diagnosis rates in both women and men have consistently been higher among 25–29-year-olds and 30–39-year-olds throughout the period compared to other age groups (2). In Italy, an evident decrease in the number of new HIV diagnoses is observed since 2018, and HIV incidence was lower compared to that reported in the European Union (3.3 new diagnoses per 100,000) (3). According to the HIV Italian Surveillance Data, the 25–29 year age group had the highest incidence of HIV infection (10.4 new diagnoses per 100,000) (3). It could be argued that testing strategies both in Europe and in Italy, are not working properly to diagnose HIV early (1). This is likely to be particularly challenging during the COVID-19 pandemic given the impact on HIV and other health and social care services.

There is strong evidence that an early diagnosis of an HIV infection and subsequent treatment can result in a markedly improved prognosis for the individual (4, 5). Moreover, the cost of treatment and care for individuals diagnosed early is significantly lower than for those diagnosed at a late stage of infection. The WHO recommends HIV testing and counseling for all patients showing signs and symptoms of the disease (6). In June 2019, the US Preventive Services Task Force (USPSTF) updated recommendations about screening and diagnostic testing for HIV infection and highlighted the effectiveness of specific preventive care services for patients without obvious related signs or symptoms (7). The USPSTF recommends that clinicians screen for HIV infection in adolescents and adults aged 15 to 65 years. Younger adolescents and older adults who are at increased risk of infection should also be screened (7). Similarly, the Centers for Disease Control and Prevention (CDC) recommends that everyone between the ages of 13 and 64 get tested for HIV at least once as part of routine health care, and for individuals at higher risk for getting HIV at least once a year (8, 9).

The assessment and identification of factors that affect the utilization of HIV testing help policymakers to design effective strategies toward preventing and controlling HIV/AIDS. According to studies conducted in different parts of the world, age of the respondent, stigmatized attitudes, levels of knowledge about HIV/AIDS and risky sexual behaviors, such as early age of sexual debut, are significantly associated with HIV testing (10, 11). Even though HIV testing is crucial for all strategies related to the care, prevention and treatment of HIV/AIDS, several studies have identified a low prevalence of HIV testing among young adults (12–14). To test the hypothesis that HIV testing is less practiced among undergraduate university students, the present study assessed the uptake and factors associated with HIV testing in a region of southern Italy.

## Materials and Methods

### Study Design and Setting

This cross-sectional study was undertaken between the 1st and 31st of July 2020, among a sample of students aged 18–29 years randomly selected from “Magna Græcia” University of Catanzaro, which is located in the southern part of Italy. The University provides medical or life sciences majors (i.e., medicine, nursing, pharmacy, dentistry, and other healthcare professions), social sciences majors (i.e., law, business economics, psychology, sociology) and technology majors (i.e., computer engineering, bioengineering).

### Sampling and Survey Instrument

After the Ethical approval obtained by the Regional Human Research Ethics Committee (ID No.102/2021703/18), data were collected using an online self-administered questionnaire. No personally identifiable information was collected. The online survey was sent via student institutional email and could only be submitted once for each electronic device to reduce potential repeat responses. Informed consent was obtained before filling out the questionnaire. There were no incentives offered for participation. The inclusion criteria required the participants to be undergraduate students. The exclusion criteria included being under 18 and over 29 years of age. The questionnaire included 24 questions divided into five sections. Each section elicited responses in a variety of formats: closed-ended questions with multiple answers possible, 5-point Likert scale options, “yes” or “no” answers and open-ended questions. The 5 sections of the questionnaire were focused on: (1) socio-demographic characteristics and sexual history of the participants; (2) knowledge regarding HIV infection (i.e. transmission route, preventive measures and testing); (3) attitudes toward HIV infection; (4) sexual and testing behaviors in the eligible participants (i.e. students who had their sexual debut). In the last section (5), the participants were asked to identify which information sources had been used to find out about HIV infection and whether they were interested in receiving additional information. Before collecting data, a pilot test was conducted to ensure question clarity, format and sequence, and minor refinements were made to improve flow and clarity.

### Statistical Analysis

Data were summarized using mean and standard deviations (SD) for continuous data and frequencies for categorical data. A logistic regression model was developed to explore the role of potential predictors of having ever tested for HIV in the eligible students. The following selected independent variables were included in the Model: gender (male = 0; female = 1), age, in years (continuous), attending to medical or life sciences majors (no = 0; yes = 1), need for further information on HIV infection (no = 0; yes = 1), knowledge score about HIV infection (ordinal) and high-risk sexual behaviors (no = 0; yes = 1). The knowledge score about HIV infection was calculated by assigning one point for each correct response and summing the scores for each statement (range 0–8). High-risk sexual behaviors has been defined as at least one sexual activity which exposes the individual to the risk of contracting HIV. The focus in the present study has been on unprotected sexual intercourse, early sexual debut (defined as having had first sexual intercourse at or before age of 14 years), alcohol consumption before sexual intercourse and having multiple sex partners (four or more people) during their lifetime (11, 15, 16). Moreover, to assess whether knowledge about HIV infection acts as a mediator of the effect of the major attended (i.e. attending medical or life sciences majors) on having ever tested for HIV, the Baron and Kenny method was used (17). The goodness of fit of the logistic regressions was ascertained through the Hosmer and Lemeshow test. The interaction between high-risk sexual behaviors and attending medical or life sciences majors was also investigated.

The statistical significance level was set at a *p*-value < 0.05. Adjusted odds ratio (OR) and 95% confidence interval (CI) were calculated.

The data were analyzed using STATA software, version 16.1 (18).

## Results

### Socio-Demographic and Sexual History Characteristics of the Participants

Among the 1,007 students who completed the survey, more than two-thirds (68.2%) were female and the median age was 23 years (IQR: 21–25 years). Of all participants, 58.8% were enrolled in medical or life sciences courses. Regarding sexual history, the mean age at first sexual intercourse was 17.5 years (SD ± 2.1), and 14.5% had experienced their sexual debut ≤ 14 years.

### Respondents' Level of Knowledge Related to HIV Infection

Only 62.6% of the students were knowledgeable that HIV can be transmitted through certain body fluids, such as blood, vaginal secretions and semen. The knowledge statements about HIV infection is detailed in Table 1. Regarding HIV testing, only 41.5 and 54.7% of the subjects knew that in Italy the test for early detection of HIV infection does not need to be prescribed by a physician and that it is provided to anyone free of charge, respectively. The vast majority (90.1%) of the students was knowledgeable that consistent condom use is a safe sexual behavior. In addition, 65.5% knew that avoiding unprotected sexual intercourse with an unknown partner and 17.4% wrongly believed that getting vaccinated for HIV before having risky relationships can prevent the sexual transmission of the infection. Furthermore, only 19.4% of the respondents knew that an individual could experience early symptoms of the acute primary infection within 2 to 4 weeks after infection with HIV and fewer than one-third (30.6%) knew that exposure to HIV and seroconversion (i.e., when the body produces enough antibodies to be detected by standard HIV testing, namely “window period”) usually ranged from 20 to 90 days. The overall median knowledge score was 4 (IQR: 3–5).

**Table 1:** Respondents' knowledge related to HIV infection.

**Statements (1007)**	***N***	**%**
**HIV can be transmitted via**		
Blood (true)	943	93.6
Sexual contact (true)	843	83.7
**HIV testing**		
Has to be prescribed by a physician (false)	418	41.5
Is provided to anyone free of charge (true)	551	54.7
**HIV transmission could be prevented by**		
Consistent condom use (true)	907	90.1
Avoid unprotected sexual intercourse with unknown partner (true)	660	65.5
Vaccine for HIV before having risky relationships (false)	175	17.4
**An individual could experience early symptoms of the acute primary infection within 2 to 4 weeks after infection with HIV** (true)	195	19.4
**The time between exposure to HIV and seroconversion (i.e., window period) usually ranged from 20 to 90 days** (true)	308	30.6

### Respondents'attitudes Toward HIV Infection and Self-Reported Sexual and Testing Behaviors

Attitudes toward HIV infection and self-reported sexual and testing behaviors are detailed in Table 2. Having multiple sex partners was perceived as a risk factor of HIV infection by 79.8% of the students. Furthermore, almost two-thirds of the participants (61.1%) believed that individuals with HIV are discriminated and stigmatized by people at large. Regarding testing behaviors, only 16.2% of the eligible students reported having ever tested for HIV and a very similar proportion (17.8%) was displayed among those who reported risky sexual behaviors. The results of the multiple logistic regression analysis indicated that the strongest predictor of having ever tested for HIV was attending medical or life sciences majors (OR: 2.32; 95% CI: 1.46–3.68). Indeed, the odds of having ever tested for HIV resulted in a 23% and 7% increase with every one-point increase in knowledge score about HIV infection (OR: 1.23; 95% CI: 1.08–1.40) and every 1 year increase of age (OR: 1.07; 95% CI: 1.00–1.77), respectively. Furthermore, having high-risk sexual behaviors (OR: 1.67; 95% CI: 1.01–2.77) was positively associated with HIV testing (Table 3). The Kenny and Baron analysis suggested that the association between having ever tested for HIV and attending medical or life sciences majors was mediated by the level of knowledge score about HIV infection. Indeed, when the knowledge score about HIV infection was removed from the regression model, the OR of attending medical or life sciences majors suggested a direct effect of that variable (0.43) on having ever tested for HIV (2.75 vs. 2.32). No interaction was shown between having high-risk sexual behaviors and attending medical or life sciences majors.

**Table 2:** Respondents' attitudes toward HIV infection and self-reported sexual and testing behaviors.

**Attitudes (1007)**					**Strongly agree/Agree**		**Uncertain**		**Strongly disagree/disagree**	
					***N***	**%**	***N***	**%**	***N***	**%**
Having multiple sex partners increases the chances of acquiring HIV					803	79.8	98	9.7	106	10.5
Individuals with HIV are discriminated and stigmatized by people at large					615	61.1	239	23.7	153	15.2
Behaviors (846)^*^	**Never**		**Rare**		**Occasionally**		**Often**		**Always**	
	***N***	**%**	***N***	**%**	***N***	**%**	***N***	**%**	***N***	**%**
Use of condom during sexual intercourse	100	11.8	80	9.5	117	13.8	165	19.5	384	45.4
Alcohol consumption before sexual intercourse	290	34.3	234	27.7	273	32.3	47	5.5	2	0.2
					**Yes**		**No**		**Mean**±**SD**	
					*N*	%	*N*	%		
Ever tested for HIV					137	16.2	709	83.8		
Number of sex partners									3.9 ± 5	
Age at first sexual intercourse									17 ± 2.1	

* *Eligible participants were those who had their sexual debut*.

**Table 3:** Results of the regression model for potential determinants of having ever tested for HIV.

***Model: Outcome: Having ever tested for HIV***			
**Variables**	**OR**	**95% CI**	***p***
**Attending medical or life sciences majors**			
No*	1.00		
Yes	2.32	1.46–3.68	<0.001
**Knowledge score on HIV infection, ordinal**	1.23	1.08–1.40	0.002
**High-risk sexual behaviors**			
No*	1.00		
Yes	1.67	1.01–2.77	0.047
**Age, continuous**	1.07	1.00–1.77	0.050
**Need further information on HIV infection**			
No*	1.00		
Yes	0.75	0.50–1.18	0.155
**Gender**			
Male*	1.00		
Female	0.88	0.59–1.31	0.526

*Log likelihood = −348.16373; Prob > chi2 < 0.001; Obs = 846*.

*(Eligible participants were those who had their sexual debut)*.

* *Reference category*.

### Respondents' Sources of Information

The main sources of information used to learn about HIV used by the students were mass media (56.5%), followed by University (54.1%), social networks (46%) and government websites and international organizations (42%). However, almost three-quarters (72.5%) of the participants wished to receive additional information.

## Discussion

This study sought to determine the uptake and factors associated with HIV testing among undergraduate university students in a region of southern Italy, given the importance of voluntary HIV testing in mitigating the impacts of HIV/AIDS, especially among young adults.

The majority of students (83.8%) in the sample had never undergone HIV testing during their lifetime, and a very similar proportion (82.2%) was displayed among those who reported risky sexual behaviors. These figures deserve consideration, since in Europe the European, Center for Disease Prevention and Control (ECDC) estimated that one in seven people living with HIV are unaware of their status (19), and in the United States 40% of new infections of HIV are transmitted by those who are not aware of their HIV diagnosis (20). In the present study, HIV testing uptake is less than that reported among adolescents in previous published studies in southern African countries (21), where progress is being made. That is to be expected, since African countries are the world's epicenter of HIV/AIDS, and most of the worst affected countries form an “AIDS belt” in eastern and southern Africa (22). Attention to this seems to be lower in Italy (23) and in western European countries, other than the United Kingdom (24). The majority of studies have been from the United States (25), aside those from Sub-Saharan Africa (26). In the latter, although patterns of HIV testing utilization vary from group to another (e.g., gender, age category) (27), a universal awareness and a very high level of HIV knowledge attributable to sustained health education programs, have been shown (28). Although in Europe the number of new HIV infections is lower than Africa, efforts to diagnose HIV early are urgently needed to curb the rising numbers of undiagnosed infections and late presenters (1). Mention must also be made of the HIV/AIDS-treatment costs. Evidence exist that the total costs for HIV positive, asymptomatic and never before on highly active antiretroviral therapy (HAART) patients were over six times lower than patients in “HAART failure” (i.e., primary HAART regimen was altered because of severe side effects or immunological failure) (29). In Italy, it has been proven that HIV/AIDS treatment costs an average of about €6,399 per patient (30).

It also should be pointed out that one time screening to identify HIV infections early is cost-effective to reduce the proportions of undiagnosed and late-diagnosed infections, even in cases of prevalence rates below 0.1% (31, 32) as well as to decrease the mother-to-child transmission (33). Repeated screening has proved useful in high-risk groups in specific settings (34). It was demonstrated that high rates of sexual risk behaviors put young people at risk for HIV (16), and sexual orientation play a significant role in the decision to undergo testing (35). Sexual orientation of the participants was not investigated in the study and future research need to deepen the distribution of lifetime HIV testing in relation to sexual orientation identity, with gay or lesbian and bisexual individuals categorized separately.

Besides, there is evidence of the public health benefit of HIV testing through the adoption of safer sexual behaviors by diagnosed individuals (36).

Another key finding of this study is that lack of knowledge related to HIV infection (e.g., transmission route and testing) is a strong predictor of uptake of HIV testing. This finding is consistent with findings from similar studies in Sub-Saharan Africa (37). Poor knowledge can fail in applying all preventive measures to reduce HIV transmission at both the individual and community levels. In particular, the fact that one third of the sample did not recognize “avoiding unprotected sexual intercourse with unknown partner” among the measures that can be taken to prevent the sexual transmission of HIV is worrying, especially among young adults, for at least a couple of reasons. First, lack of knowledge about conditions of possible exposure to HIV can result in a missed opportunity to ask for a diagnostic HIV test and, ultimately, in a late presentation for HIV care. Second, this figure could have an impact on the use of one effective pharmacological prevention tool available, namely post-exposure prophylaxis (PEP). PEP is a course of antiretroviral medicines that can prevent HIV after an event that might have put an individual at risk of infection, such as having had sex without a condom with someone of unknown HIV status (38), a situation that could be common among young adults. A previous study showed that the comprehensive knowledge of role and timing (i.e., within 72 h of possible exposure) of PEP was weak and poor among college students (39). In addition, less than one fifth of participants, knew that primary symptoms of HIV infection may show up 2 to 4 weeks after initial exposure. As per evidence-based recommendations, patients should be screened for HIV if they present with signs and symptoms of HIV (40). However, it is unlikely that an individual would ask for a HIV test if he/she is unaware that a constellation of non-specific symptoms, such as fever, sore throat, or rash, may present 2 to 4 weeks after potential exposure to HIV transmission. Both these results could represent an issue of major concern since there is strong evidence that an early diagnosis of an HIV infection and subsequent antiretroviral therapy (ART) can result in a markedly improved prognosis for the individual who can expect low morbidity, a good quality of life, and a near normal life expectancy. Indeed, individuals with HIV who take HIV drug as prescribed and maintain an undetectable viral load have effectively no risk of transmitting HIV to HIV-negative sex partners (1). These findings contribute in identifying gaps in HIV infection knowledge and could help to design effective strategy toward HIV control and prevention. It is well-known that several organizations (41–43) recommended implementing prevention strategies as a key factor to tackle HIV transmission, with the adoption of a combination of preventive interventions, such as frequent testing, access to condoms, pre-exposure prophylaxis and counseling. Preventive services should guarantee the data privacy and be free, with a friendly approach, especially when targeted young people who should feel free to ask questions about sexual health.

The finding that almost half of the sample did not know that HIV testing can be provided to anyone free of charge and without a prescription of a physician underscore the need to train both primary care providers, so that they can counsel their patients and recommend HIV testing, and public health professionals to become aware of individual's health knowledge needs.

Understanding provider attitudes and practices as potential barriers to routine HIV testing represent an important area of research, since training healthcare providers can effectively increase HIV testing rates (38). HIV testing needs to be incorporated into every level of the healthcare system to diagnose HIV as early as possible, and all healthcare providers should be aware of the screening recommendations (44).

Attention should also be given to public health literacy as an important element of individual-centered care. Given healthcare's rapid changes and speed at which technology is progressing, there is a need to support more regular updates of knowledge and maintain the health skills of individuals throughout life (45), using all potential resources (e.g., social media). In accordance with previous studies in the same area (46, 47), social networks and government websites were reported as major sources of health information. Therefore, health organizations have to consider social media and the Internet within their communication strategy to promote the appropriate Web use for HIV-related information seeking, especially among young people who are the most digitally savvy population group (45, 48). Moreover, policy-makers should consider including sexual education in the high-school curricula since lasting and multi-component school-based interventions can improve HIV knowledge and attitudes (49, 50).

Lastly, it could be mentioned that, during the first wave of the COVID-19 pandemic, some community-based organizations and health clinics ceased to offer in-person evaluations and HIV screening (51) resulting in a decreasing number of new HIV diagnoses reported in Italy in 2020 (2.2 per 100,000 residents), lower compared to that reported in the previous year (52).

### Limits

Certain potential methodological limitations should be considered in the interpretation of the findings from this study. First, the cross-sectional design prevents drawing conclusions regarding causality and temporal sequence and, therefore, this study can only describe general associations. Nonetheless, the results of this study can give an update on the uptake of HIV testing among university students in southern Italy. Second, one should be cautious when generalizing the findings because this survey was limited to students in one university in southern Italy, thus the results might not truly reflect the knowledge and the attitudes of the whole population of Italian students. Nevertheless, we are confident that the data can be representative at least of the university students in this part of the country. Lastly, one cannot rule out that there could be a tendency by the respondents to over-report desirable attitudes or behaviors (e.g., sexual) and/or to under-report socially undesirable attitudes or behaviors as a manifestation of social desirability. However, to mitigate this bias and improve the accuracy of the information we guaranteed the participants confidentiality and anonymity and assured that the data obtained from the study were for research purposes only.

## Conclusion

The results of the present study highlighted that effective strategies to increase HIV testing and diagnoses have to aim at overarching individual barriers such as lack of knowledge about information around the test itself. To address this issue, the role of the Ministry of Health and national governmental institutions is pivotal to increase public awareness on the causes and prevention of HIV/AIDS, and to allocate resources to implement HIV testing policies and programs. Mass media campaigns can have an impact on testing behaviors, with most HIV testing campaigns successfully increasing HIV testing rates (52) and decreasing the proportion of individuals living with HIV who are unaware of their status.

## Data Availability Statement

The datasets presented in this study can be found in online repositories. The names of the repository/repositories and accession number(s) can be found below: Mendeley Data repository (doi: 10.17632/pwcpbhysv7.1)

## Ethics Statement

The study was conducted according to the guidelines of the Declaration of Helsinki, and approved by the local Human Research Ethics Committee (protocol No.102/2021703/18). The patients/participants provided their written informed consent to participate in this study.

## Author Contributions

FL and SA participated in the conceptualization and in the design of the study. FL and CGAN contributed to the data collection. FL, SA, and GDG contributed to the data analysis and interpretation. FL, SA, and GDG contributed to the data analysis, interpretation, and preparation of the first draft of the manuscript. CGAN and AB were responsible for funding acquisition and resources provision. AB, the principal investigator, designed the study, coordinated and supervised data collection, was responsible for the statistical analysis and interpretation, and wrote the final article. All the authors have given final approval of the version to be published and agreed to be accountable for all aspects of the work.

## Conflict of Interest

The authors declare that the research was conducted in the absence of any commercial or financial relationships that could be construed as a potential conflict of interest.

## Publisher's Note

All claims expressed in this article are solely those of the authors and do not necessarily represent those of their affiliated organizations, or those of the publisher, the editors and the reviewers. Any product that may be evaluated in this article, or claim that may be made by its manufacturer, is not guaranteed or endorsed by the publisher.
